# Supplementary material for: Proteome and Secretome Dynamics of Human Retinal Pigment Epithelium in Response to Reactive Oxygen Species
Source: Sci Rep. 2019 Oct 28;9:15440. doi: 10.1038/s41598-019-51777-7 (PMC6817852; doi:10.1038/s41598-019-51777-7)
Supplement: Supplementary file 1 — Supplemental Figures [file 41598_2019_51777_MOESM1_ESM.docx]

**Proteome and Secretome Dynamics of Human Retinal Pigment Epithelium in Response to Reactive Oxygen Species**

**Authors:**

Jesse G. Meyer^1,2^*, Thelma Y. Garcia^1^, Birgit Schilling^1^, Bradford W. Gibson^1,3^, Deepak A. Lamba^1,4^*

**Affiliations:**

^1^ Buck Institute for Research on Aging, Novato, CA, 94945, USA.

^2^ Current Address: Department of Chemistry, Department of Biomolecular Chemistry, National Center for Quantitative Biology of Complex Systems, University of Wisconsin - Madison, Madison, WI, 53706, USA.

^3^ Current Address: Discovery Attribute Sciences, Research, Amgen, South San Francisco, CA 94080, USA

^4^ Current Address: Department of Ophthalmology, Eli and Edythe Broad Center of Regeneration Medicine and Stem Cell Research, University of California - San Francisco, San Francisco, CA, 94143, USA.

*Correspondence to: [jessegmeyer@gmail.com](mailto:jessegmeyer@gmail.com) or [Deepak.Lamba@ucsf.edu](mailto:Deepak.Lamba@ucsf.edu)


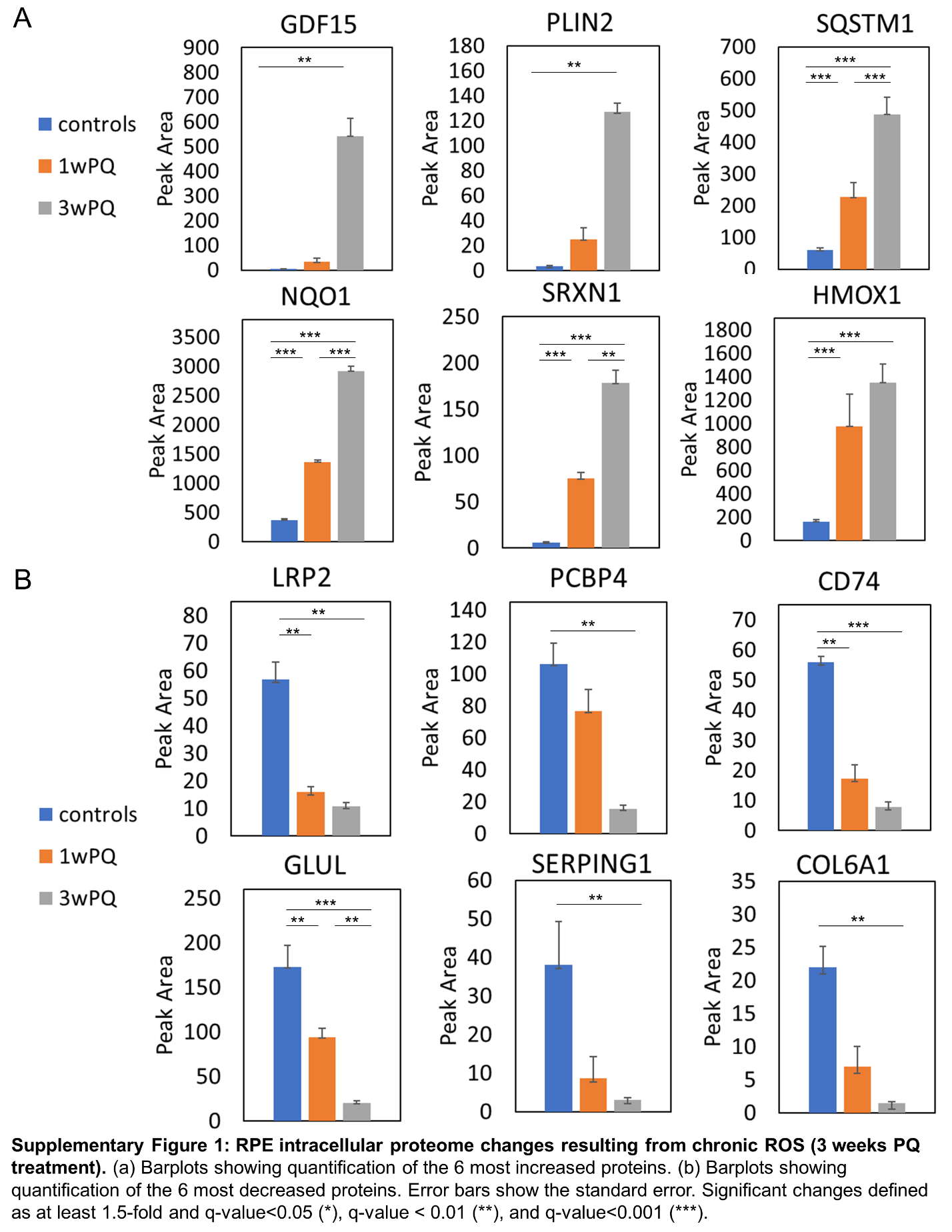


**Supplementary Figure 1: RPE intracellular proteome changes resulting from chronic ROS (3 weeks PQ treatment).** (a) Barplots showing quantification of the 6 most increased proteins. (b) Barplots showing quantification of the 6 most decreased proteins. Error bars show the standard error. Significant changes defined as at least 1.5-fold and q-value<0.05 (*), q-value < 0.01 (**), and q-value<0.001 (***).


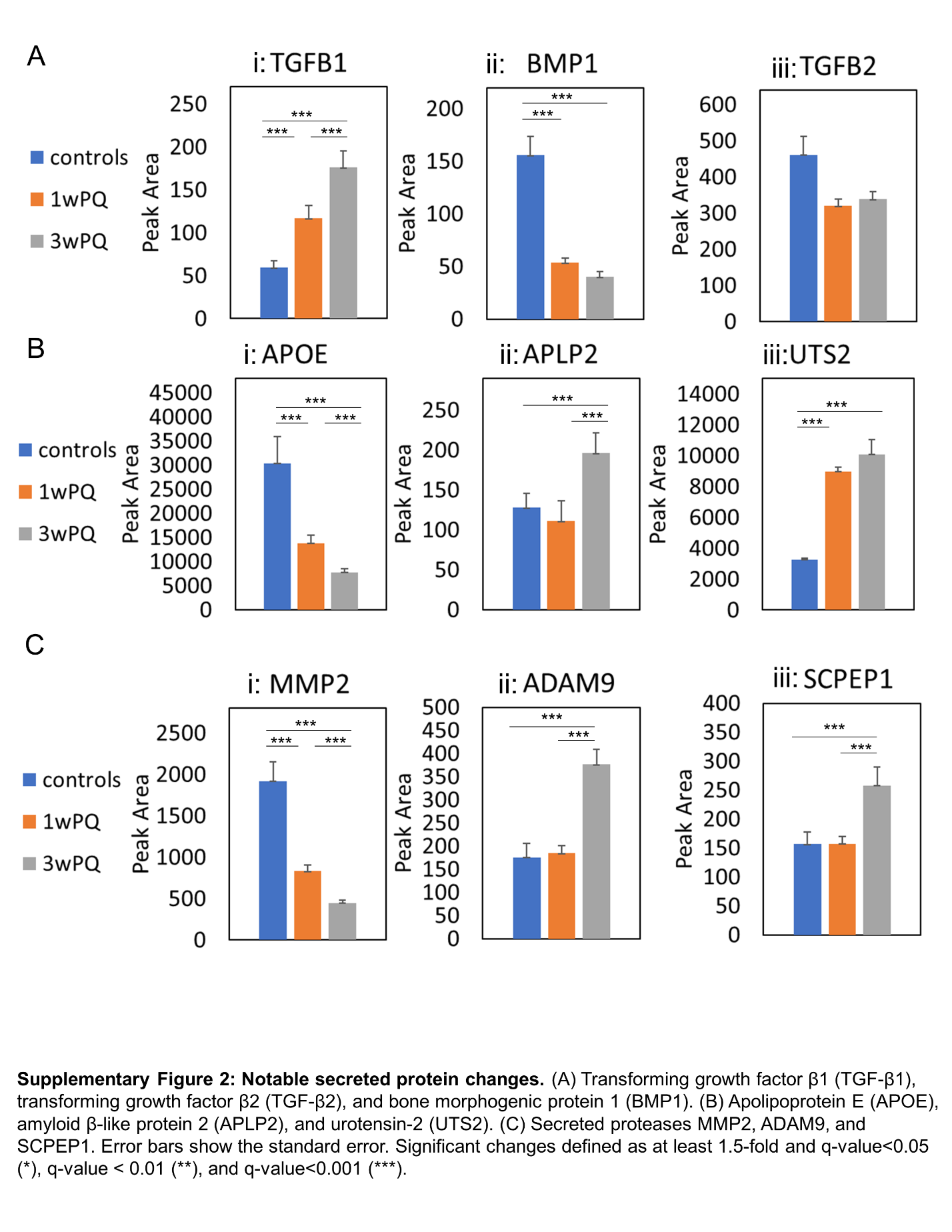


**Supplementary Figure 2: Notable secreted protein changes.** (A) Transforming growth factor β1 (TGF-β1), transforming growth factor β2 (TGF-β2), and bone morphogenic protein 1 (BMP1). (B) Apolipoprotein E (APOE), amyloid β-like protein 2 (APLP2), and urotensin-2 (UTS2). (C) Secreted proteases MMP2, ADAM9, and SCPEP1. Error bars show the standard error. Significant changes defined as at least 1.5-fold and q-value<0.05 (*), q-value < 0.01 (**), and q-value<0.001 (***).
